# Severe Pollution in China Amplified by Atmospheric Moisture

**DOI:** 10.1038/s41598-017-15909-1

**Published:** 2017-11-17

**Authors:** Xuexi Tie, Ru-Jin Huang, Junji Cao, Qiang Zhang, Yafang Cheng, Hang Su, Di Chang, Ulrich Pöschl, Thorsten Hoffmann, Uli Dusek, Guohui Li, Douglas R. Worsnop, Colin D. O’Dowd

**Affiliations:** 1 Key Laboratory of Aerosol Chemistry and Physics, Institute of Earth Environment, Chinese Academy of Sciences, Xi’an, 710061 China; 2 State Key Laboratory of Loess and Quaternary Geology, Institute of Earth Environment, Chinese Academy of Sciences, Xi’an, 710061 China; 3 Center for Excellence in Urban Atmospheric Environment, Institute of Urban Environment, Chinese Academy of Sciences, Xiamen, 361021 China; 4 National Center for Atmospheric Research, Boulder, CO USA; 5 School of Physics and Centre for Climate and Air Pollution Studies, National University of Ireland Galway, Galway, Ireland; 6 Beijing Weather Modification Office, Beijing, China; 7 Multiphase Chemistry Department, Max Planck Institute for Chemistry, Mainz, Germany; 8 Institute of Inorganic and Analytical Chemistry, Johannes Gutenberg University of Mainz, Duesbergweg 10–14, 55128 Mainz, Germany; 9 Centre for Isotope Research (CIO), Energy and Sustainability Research Institute Groningen (ESRIG), University of Groningen, Groningen, The Netherlands; 10 Aerodyne Research, Inc, Billerica, MA USA

## Abstract

In recent years, severe haze events often occurred in China, causing serious environmental problems. The mechanisms responsible for the haze formation, however, are still not well understood, hindering the forecast and mitigation of haze pollution. Our study of the 2012–13 winter haze events in Beijing shows that atmospheric water vapour plays a critical role in enhancing the heavy haze events. Under weak solar radiation and stagnant moist meteorological conditions in winter, air pollutants and water vapour accumulate in a shallow planetary boundary layer (PBL). A positive feedback cycle is triggered resulting in the formation of heavy haze: (1) the dispersal of water vapour is constrained by the shallow PBL, leading to an increase in relative humidity (RH); (2) the high RH induces an increase of aerosol particle size by enhanced hygroscopic growth and multiphase reactions to increase particle size and mass, which results in (3) further dimming and decrease of PBL height, and thus further depressing of aerosol and water vapour in a very shallow PBL. This positive feedback constitutes a self-amplification mechanism in which water vapour leads to a trapping and massive increase of particulate matter in the near-surface air to which people are exposed with severe health hazards.

## Introduction

Similar to that previously experienced by the developed nations, rapid industrialization and urbanization in China has led to an increase in air pollution. As the world’s largest developing country, China has experienced severe haze pollution in the past two decades. In large cites of China, heavy haze episodes often occurred in recent years, For example, during the 2012–13 winter, severe haze events were frequently observed in Beijing, China. In this period, the hourly PM_2.5_ concentrations frequently exceeded 200 μg m^−3^. The extremely high aerosol concentrations led to very low visibility, especially during December 10 to 15 (less than 1 km). Such high concentrations of PM_2.5_ can cause serious adverse effects on human health and welfare^1–6^. The temporal and spatial features and causes of heavy haze formation are, however, not well understood, leading to a lack of efficient control and mitigation strategies.

## Characteristic of Haze Episodes

**Figure 1:**
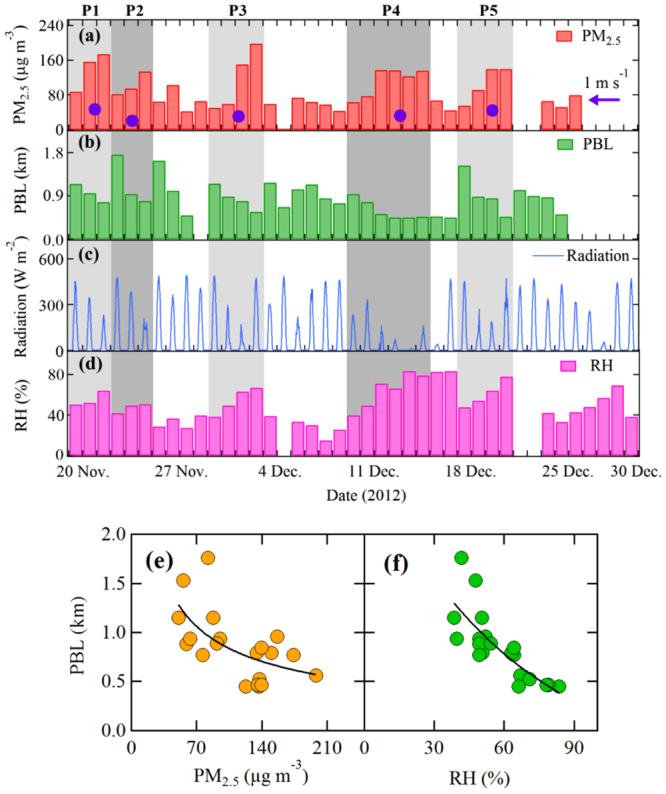
Temporal variations of aerosol particle concentration, planetary boundary layer (PBL) height, solar radiation and relative humidity (RH) during 2012–2013 winter time in Beijing. Measured (**a**) daily averaged PM_2.5_ concentrations, (**b**) daytime (8:00–18:00) PBL heights, (**c**) solar radiation, and (**d**) RH from Nov. 21 to Dec. 30, 2012. Five episodes with remarkable features of the heavy haze events were found, referred to as P1 (Nov. 21–23), P2 (Nov. 24–26), P3 (Nov. 30-Dec. 4), P4 (Dec. 11–16), and P5 (Dec. 19–22). The purple dots show the averaged wind speeds. The purple arrow marks the 1 m/s position. Correlations between PBL heights and PM_2.5_ concentrations (**e**) and between PBL heights and RH (**f**) during the five haze episodes.

Figure 1(a–d) Illustrates the daily averaged PM_2.5_ concentrations, daytime (8:00–18:00) mean planetary boundary layer (PBL) heights, relative humidity (RH), and daily variation of solar radiation from November 20 to December 30, 2012. The details about the measurements and instruments are described in previous studies^7,8^ (also see Supplementary Information). Five episodes during this time period with remarkable features of the heavy haze events were identified as P1 (Nov. 21–24), P2 (Nov. 25–27), P3 (Nov. 29-Dec. 2), P4 (Dec. 10–15), and P5 (Dec. 19–22). They are characterized by continuously increasing PM_2.5_ concentrations and high RH, with daily averaged PM_2.5_ concentrations exceeding 100 μg m^−3^. For example, the daily mean concentration of PM_2.5_ was higher than 200 μg m^−3^ at the end of P3, and for P1, P2, P4, and P5, the highest concentrations ranged from 135 to 175 μg m^−3^. Corresponding to the rapid increase in PM_2.5_ concentrations, the daytime mean PBL heights decreased significantly, from 0.8 to 1.1 km at the beginning of the five episodes to 0.6 to 0.4 km at the end. As shown in Fig. 1e,f, not only the PM_2.5_ concentration but also the RH value was strongly anti-correlated with the PBL height, which suggests that the shallow PBL heights indeed suppressed the dispersal of water vapour. This is further confirmed by the *in-situ* aircraft measured vertical profiles of PBL heights, aerosol particles, RH, and water vapour (see Figure S1 of Supplementary Information). As discussed below, the accumulation of water vapour in the shallow boundary layer plays a key role in a self-amplification mechanism in the development of heavy haze. It is important to note that the evolution of a haze episode involves many meteorological and chemical factors, such as wind direction, wind speed, PBL height, humidity, chemical reactions, etc. These factors are non-linearly correlated or anti-correlated, with a very complicated relationship. As shown in Fig. 1e, there is a large dispersion of the relationship between PM_2.5_ and PBL, suggesting a complicated relationship by involving these different factors.

## Evolution of PBL, Aerosol, Water Vapour, and Solar Radiation

It is worth noting that during these five episodes, the measured surface wind speeds were all very low (less than 1 m s^−1^, marked by the purple dots in Fig. 1a), indicating a stagnant weather condition and a very weak horizontal dispersion (transport) of aerosol particles. Under such conditions, the vertical diffusion and the PBL heights usually play important roles in controlling the variability of aerosol particles^9^. As shown in Figure S1, the decrease in the PBL heights compressed both the air pollutants and water vapour into a shallow vertical layer, enhancing the aerosol concentrations and relative humidity near the ground surface.

One of the important reasons for the decrease in the daytime PBL heights was weakened solar radiation. Many previous studies indicated that the thermal turbulence caused by the surface heating of solar radiation is the major reason for a fully developed diurnal variation of the PBL heights^9–11^. A typical PBL development is that the PBL height is low (around 100 meters) in the night-time, and is increasing rapidly in the morning due to heating of the ground by solar radiation. The noontime PBL heights can reach a maximum of 1–3 km depending on locations and seasons^12–14^. As a result, it is not surprising that the daytime PBL heights were strongly correlated with the solar radiation as shown in Fig. 1b,c. For example, for P1 and P2, the noontime maximum of solar radiation was above 500 W m^−2^ in the beginning and reduced to ~200 W m^−2^ at the end of the two episodes, which led to the decrease in the PBL heights from 1.2–1.8 km to 0.7–0.8 km.

**Figure 2:**
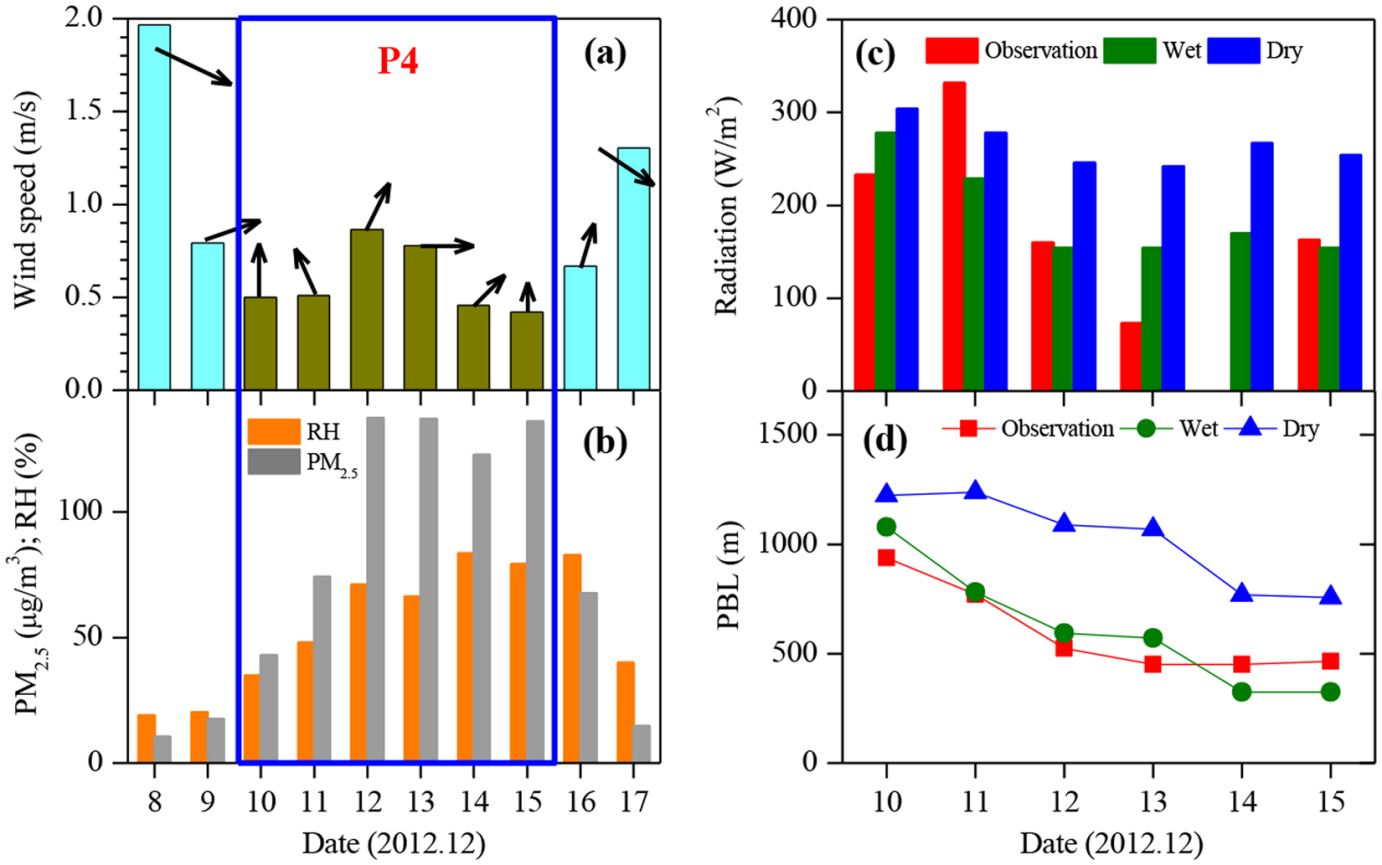
A case study of the effects of water vapour on solar radiation, PBL heights, and PM_2.5_ concentrations. (**a**) shows the measured wind speeds (m s^−1^) prior to P4, during P4 and after P4. (**b**) shows the measured PM_2.5_ concentrations (μg m^−3^) and RH (%) prior to P4, during P4 and after P4. (**c**) shows the measured and modeled (“dry” and “wet” cases) surface solar radiation during P4. (**d**) shows the measured and modeled (“dry” and “wet” cases) PBL heights. The results suggest that water vapour plays an important role in the reduction of solar radiation and PBL heights.

To illustrate the relationships among PM, water vapour, solar radiation, and PBL height, we conducted a case study to investigate the detailed interactions of these parameters. The P4 was selected for the case study, because it had persistent haze period from Dec. 10 to 15, 2012. During the period, the aerosol and water vapour concentrations were very high, leading to extremely low visibility ( < 2 km) in ~4 days. As shown in Fig. 2a, prior to the P4 period, the wind directions were northwest, with a wind speed of 2 m s^−1^ on Dec. 8, 2012. In the northwest area of Beijing, the topography is covered by mountains and grasslands, with a small population. According to previous^15,16^, under the northwest wind condition (wind speeds are often higher than 1 m/s during non-haze episodes), the northwest wind transports clean air to Beijing, resulting in low PM_2.5_ pollutions in Beijing. As shown in Fig. 2b, the PM_2.5_ concentrations were ~10 μg m^−3^. On Dec 10, 2012, the wind direction changed from northwest to south. The south winds enhanced the horizontal transport from high emission regions to Beijing, resulting in the increase in PM_2.5_ concentrations. Because there are mountains in the west and north sides of Beijing, the southern pollution plumes were blocked by the mountains, leading to an accumulation of PM_2.5_ concentrations in Beijing. As a result, the PM_2.5_ concentration rapidly increased to 50 μg m^−3^ on Dec 10. As shown in Fig. 2b, with the persistent south winds in P4, the PM_2.5_ concentrations continuously increased, and the RH values were also quickly enhanced. Figures 1 and 2 also showed that during the P4 period, the wind speeds were small (less than 1 m s^−1^), and the PBL heights decreased from 1 km to 0.5 km, with low solar radiation. At the end of the P4 period, the wind direction changed to northwest wind, and the wind speed increased, resulting in a decrease in PM_2.5_ concentrations.

## Model Calculations of Water Vapour Effect on Solar Radiation and PBL

In order to estimate the effect of PM and water vapour on solar radiation and PBL height, a state-of-the-art solar radiation model, Tropospheric Ultraviolet-Visible Model (TUV)^17,18^ and an empirical PBL model^19^ are applied in this study (the detailed methods of the two models are shown in Supplementary Information). The effects of water vapour on the solar radiation and the evolution of the PBL height are investigated in two different scenarios (a “wet” and a “dry” case), which represent different aerosol conditions, i.e., with and without the hygroscopic water uptake in the calculation of aerosol optical properties. The calculation showed that in “dry case’ (the blue bars in Fig. 2c), the calculated noontime solar radiations were ~320 W m^−2^ on Dec. 10 (at the beginning of the heavy haze), and reduced to ~275 W m^−2^ on Dec. 15 (at the end of the heavy haze), which was ~14% reduction of the surface solar radiation. However, this calculation still strongly deviates from the measured values, and cannot explain the large reduction of the measured surface solar radiation (see Fig. 2c). In contrast, when the hygroscopic process was taken into account in the calculation (‘wet case’), the simulated surface solar radiation reduced from ~290 W m^−2^ to ~160 W m^−2^ (~45% reduction) from the beginning to the end of the heavy haze event. This is consistent with the measured trends, i.e., about 240–330 W m^−2^ at the beginning and ~110 W m^−2^ at the end of P4 (~45% reduction). It suggests that the rapid increase in the RH value from Dec. 12 (RH ~50%) to Dec. 13 (RH ~80%) amplified the rapid decrease in the solar radiation, which impeded the development of PBL and favoured the further accumulation of pollutants and water vapour.

As shown in Fig. 2d, the measured daytime averaged PBL heights (red line and dots) were ~940 meters at the beginning and quickly reduced to 466 meters at the end of the heavy haze event (~50% reduction). The calculated variability of PBL heights was smaller without considering the effect of humidity (the “dry” case). At the beginning of the haze period, the calculated PBL height was ~1200 m. This value decreased to ~760 m at the end of haze period (~36% reduction), but still significantly overestimated the measured PBL height. In contrast, the calculated PBL height in the “wet” case had a much better representation. With the increase in the humidity (RH values changed from 39% on Dec. 10 to 83% on Dec. 15), the calculated PBL height decreased from 1080 m to 325 m, which was close to the measured change. This result suggested that solar radiation was largely scattered by particles and uptaken water^20,21^, and hence less solar radiation reached the ground surface (“dimming effect”), causing an unfavourable condition for a full development of the PBL. The lower PBL height further compressed/trapped the aerosol particles and water vapour in a shallow vertical layer, resulting in an even higher RH and aerosol concentration. As illustrated in Fig. 1, under low RH conditions (RH < 50–60%), PBL heights were generally greater than 700 m, while under high RH conditions (RH > 60%), the PBL heights varied between 300 and 700 m.

## Diurnal Variation During Heavy Haze Periods

**Figure 3:**
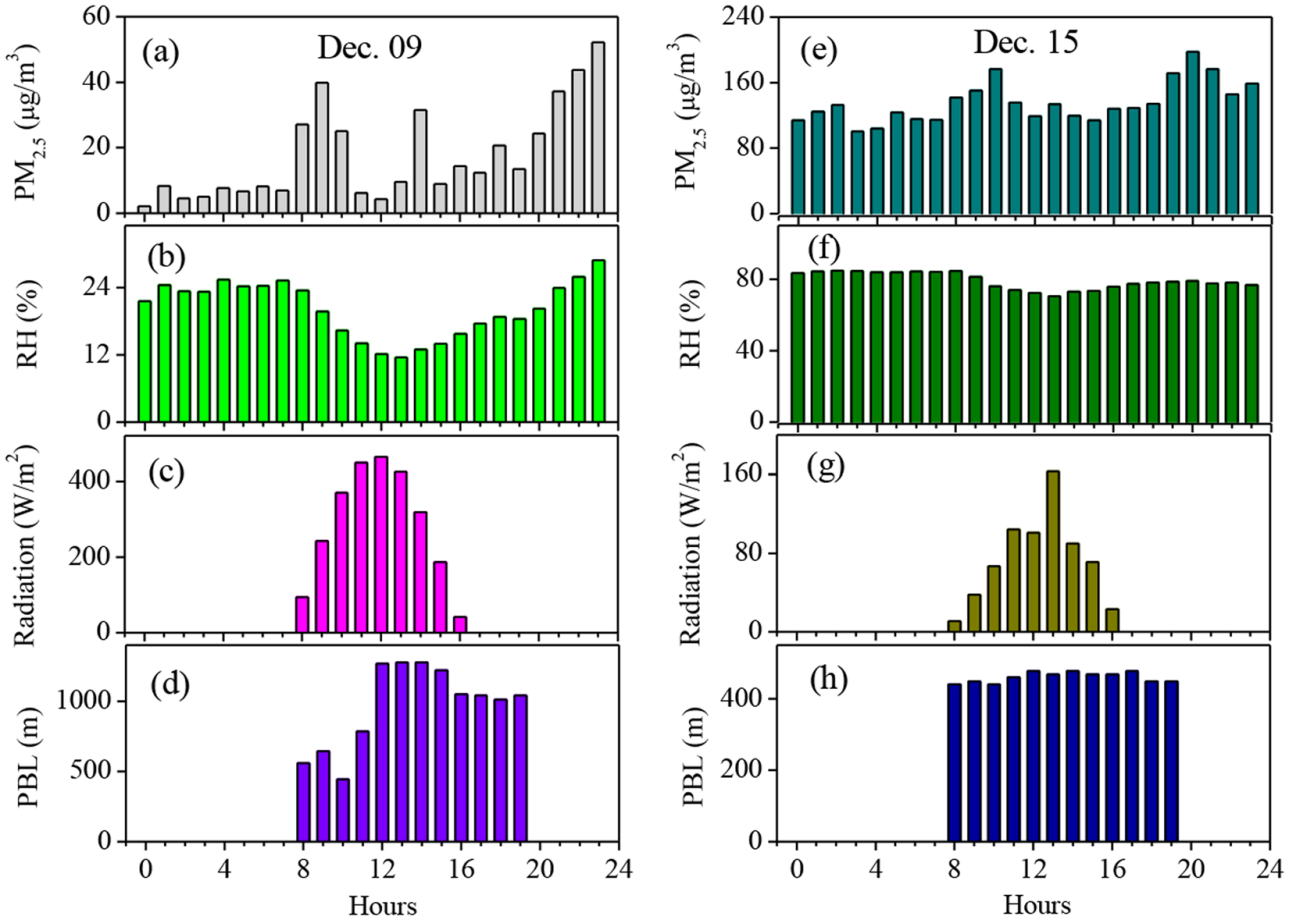
Diurnal variations of PM_2.5_ concentrations (μg m^−3^), RH (%), surface solar radiation (W m^−2^), and PBL height (km). The left column (**a**–**d**) shows the diurnal variation prior to the haze period (on Dec. 9, 2012), and the right column (**e**–**h**) shows the variation during the haze period (on Dec. 15, 2012). The results showed that there were significant diurnal variations during the non-haze period and small variations during the haze period.

Figure 3 shows the diurnal variations of PM_2.5_, RH, surface solar radiation, and PBL height. The results show that prior to the haze period (on Dec. 9, 2012), there were significant diurnal variations for PM_2.5_, RH, surface solar radiation, and PBL height. For example, the noontime solar radiation reached 460 W m^−2^ and reduced to 40 W m^−2^ at 18:00. The PBL height was only 0.5 km at 8:00, and increased to 1.3 km at 14:00. These strong diurnal cycles produced significant PM_2.5_ and RH diurnal variations. For example, there were high values of PM_2.5_ and RH in the morning due to the low PBL height, and the values of PM_2.5_ and RH rapidly decreased in the noontime, due to the increase in the PBL heights. By contrast, in the high polluted day (Dec. 15, 2012), there were non-significant diurnal variations for PM_2.5_, RH, and PBL height. This was due to the fact that the driving force of diurnal variation (solar radiation) was significantly weaker during haze period than the non-haze period. Figure 3g shows that the maximum of solar radiation was only 100–170 W m^−2^ on Dec. 15, which was much weaker than 460 W m^−2^ on Dec. 9.

## Effect of Water Vapour on Secondary Aerosols

**Figure 4:**
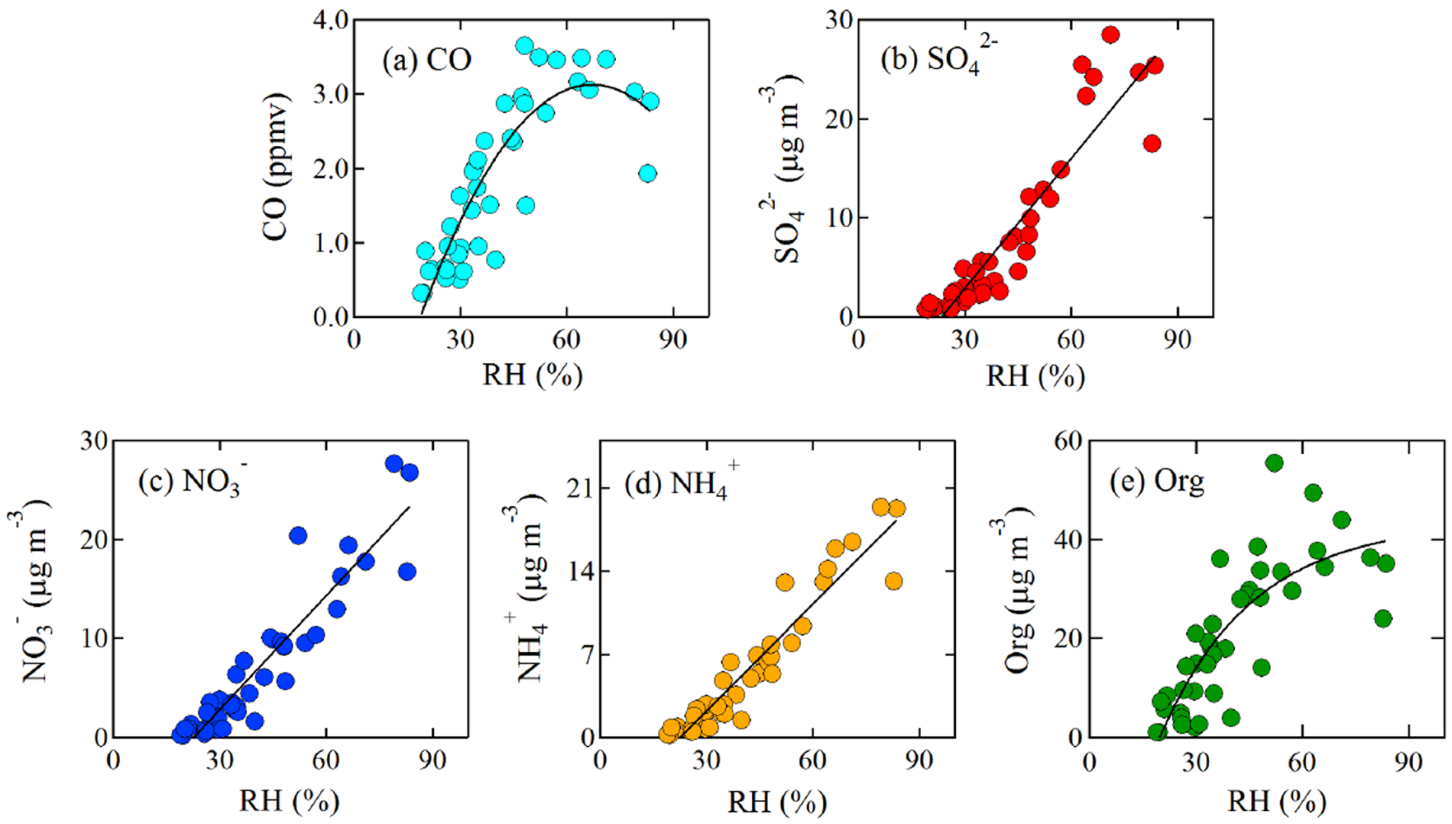
The variability of measured CO and aerosol composition (SO_4_^2−^, NO_3_^−^, NH_4_^+^, Organics) under different RH conditions during 2012–2013 winter time in Beijing. As RH increased to a critical value (50%), the CO, which can be considered as an inactive-chemical tracer, remained at a relatively constant value. The secondary aerosols (NO_3_^−^, SO_4_^2−^, NH_4_^+^), however, showed a rapid increase with the increase in RH values. The organic aerosol (Org) contained both secondary and primary component, resulting in a mixed behaviour between the CO and secondary aerosols.

Field measurements revealed that the aerosol particles during the 2012–13 winter in Beijing contained a large amount of hydrophilic aerosol particles, such as sulphate, nitrate, and ammonium^22–24^. Under high RH (>60–80%), the volume of aerosol particles can be doubled by absorbing water vapour onto the surface of aerosol particles^25,26^. The enlarged aerosol surfaces/volumes lead to more rapid multiphase reactions and secondary aerosol formation, resulting in elevated aerosol concentrations^22,27^. Simultaneous measurements of aerosol chemical composition with an Aerodyne Compact Time-of-Flight Aerosol Mass Spectrometer (C-ToF-AMS) confirm this mechanism. As shown in Fig. 4, when RH increased to a critical value (~60%), the concentrations of CO, which can be considered as an inactive-chemical tracer (i.e., it has no aqueous phase formation), remained at a relatively constant value. The primary aerosol (chloride) had the similar behavior as CO. The secondary aerosols (sulphate, nitrate, and ammonium), however, showed a rapid increase with the increase in RH values. For example, when the RH values increased from 60% to 80%, the sulphate, nitrate, and ammonium concentrations increased from 16 to 25 μg m^−3^, 15 to 23 μg m^−3^, and 11 to 17 μg m^−3^, respectively. The different trends between CO, primary and secondary aerosols suggest enhanced formation of secondary aerosols under high RH conditions. The organic aerosol contained both secondary and primary components, showing some weaker growth than the solo secondary aerosols under high RH. However, although the measurement shows that the formation of secondary aerosols rapidly grows under high humidity condition, the current understanding of chemical formation cannot explain this fast growth^28,29^. Several studies attempts to propose new chemical mechanisms to explain this issue. For example, Chen *et al*.^28^ suggest that sulfate aqueous phase formation can be enhanced by adding NO_2_ species. Wang *et al*.^29^ propose that in addition to adding NO_2_, high concentrations of NH_3_ in eastern China can significantly increase the sulfate aqueous phase formation. The scientific base of this reaction is that high levels of NH_3_ have been suggested to elevate ambient particle pH levels to near neutral acidity (pH = 7), a condition that promotes rapid SO_2_ oxidation. However, a more recent study by Guo *et al*.^30^ argues that particle pH, regardless of ammonia levels, is always acidic even for the unusually high NH_3_ levels found in Beijing (pH = 4.5) and Xi’an (pH = 5), locations where sulfate production from NO_x_ is proposed. These augments suggest that the current understanding of aqueous phase reactions, which promote the fast growth of secondary aerosols, exists a large uncertainty, which needs to be further study.

## Amplification Mechanism of Haze by Water Vapour

**Figure 5:**
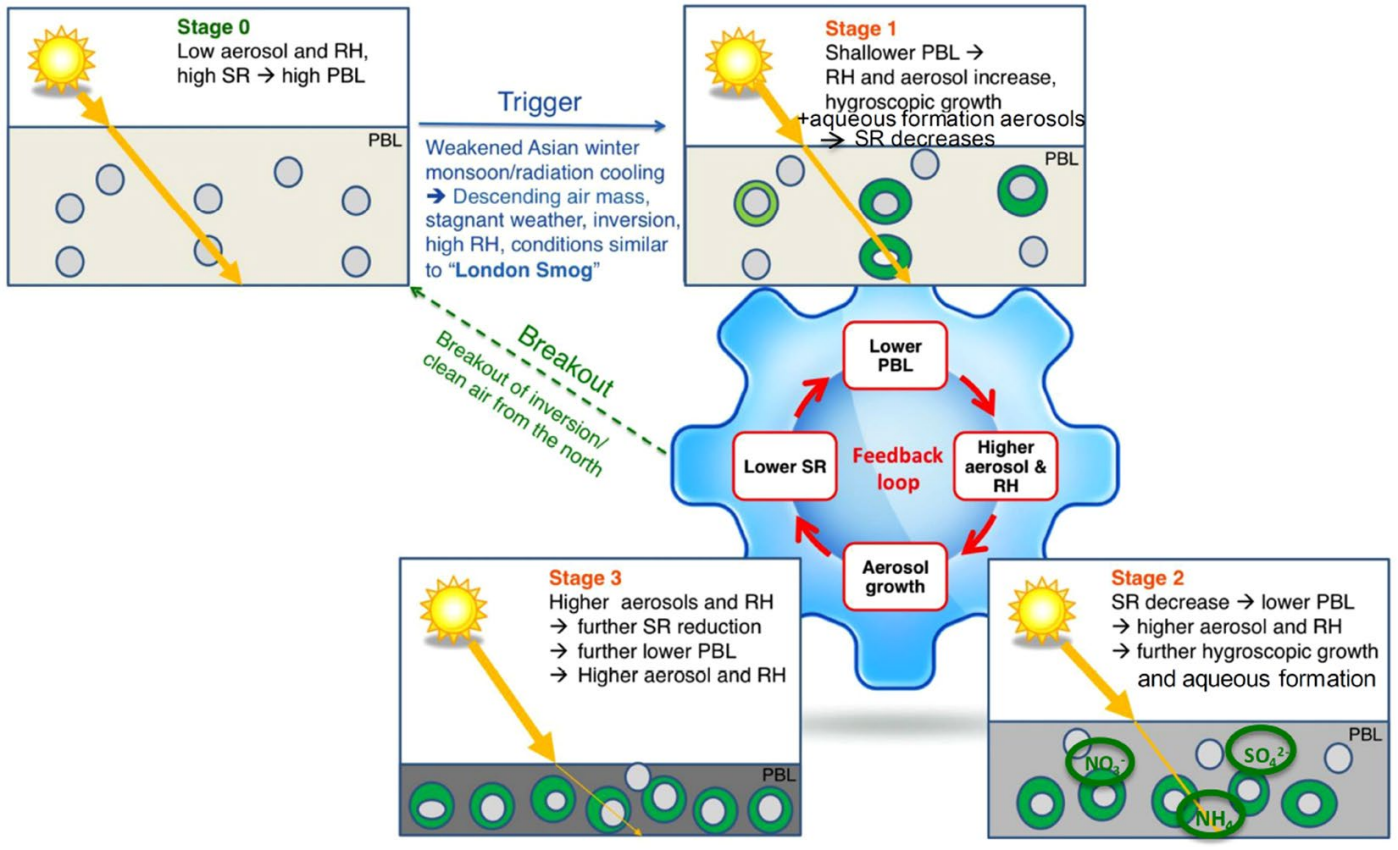
Feedback mechanism amplifies the formation of heavy haze events by aerosol uptaken water. At the beginning of the heavy haze events, the Beijing region was under northwest wind condition, the aerosol concentrations and relative humidity (RH) are low, and strong solar radiation (SR) produces high daytime planetary boundary layer (PBL) height (Stage 0). Triggered by South wind condition, aerosol pollutant and water vapour increased, leading lower solar radiation and PBL heights. With the increase in RH value, the size of aerosol particles rapidly grows and secondary aerosol forms, resulting in a stronger dimming effect near the ground surface (Stage 1). The reduction of SR depresses the development of the PBL heights, enhancing the surface aerosol concentrations and RH, which leads to further shallower PBL forming a feedback loop (stage 2). The higher aerosol concentrations and increased RH value further decrease the surface solar radiation, producing the increase in aerosol concentrations in a further shallower PBL (stage 3).

From the above discussion and analysis, we propose a self-amplification mechanism of the heavy haze formation by water vapour, which may further accelerate the haze formation and strengthen the persistency of the heavy haze conditions. A schematic plot of the proposed mechanism is illustrated in Fig. 5. Prior to the heavy haze events (Stage 0), the meteorological conditions (northwest wind, with a relatively high wind speed) produced low aerosol concentrations and humidity. Strong solar radiation produced high daytime PBL heights (e.g., at the beginning of P3 and P4, RH < 50%, PBL height ~1 km, and PM_2.5_ ~50 μg m^−3^). The stagnant inversion conditions triggered by the large weather conditions and radiation cooling leads to the increase in RH values and accumulation of air pollutants (Stage 1). Under such conditions, aerosol particles undergo enhanced hygroscopic growth and multiphase reactions and scatter more lights (Stage 2), resulting in a reduction in the surface solar radiation (in the middle of P3 and P4, RH > 60%, and solar radiation < 200 W m^−2^). This depresses the development of the PBL, leading to higher aerosol concentrations and RH values in a shallow PBL (at the end of P3 and P4, PBL height ~0.5–1.0 km, and PM_2.5_ > 100–200 μg m^−3^, RH > 70–80% throughout the PBL). The dimming effect is further amplified by the higher aerosol concentration and increased RH values, thus forming a positive feedback loop, which enhances a trapping and massive increase of particulate matter in the near-surface air (Stage 3). The key process of this feedback loop is the hygroscopic growth of aerosol particles under high humidity condition, which triggers the self-amplification feedback cycle leading to more severe haze pollution than expected.

## Conclusions

In general, our measurement and modeling results of the 2012–13 winter haze events in Beijing, China suggest that water vapour plays a critical role in the heavy haze events through a self-amplification mechanism. Such positive feedback accelerates the formation and strengthens the persistency of heavy haze events. This result has a very important implication for the heavy haze control strategy, i.e., under stagnant and high RH conditions, aggressive control measures of PM_2.5_ and precursors (NO_x_, SO_2_, NH_3_, and volatile organic compounds^23^) would be required to mitigate the wintertime heavy haze events in Beijing. The self-amplification mechanism may also occur in other heavily polluted regions (e.g., India) where a similar emission control strategy should be taken.

## Methods

This study includes *in-situ* surface measurements, aircraft measurements, and numerical model simulations. The surface measurement was conducted from Nov. 19, 2012 to Jan. 15, 2013 at Baolian meteorological station (39°56′N, 116°17′E) in the urban area of Beijing. The mass concentrations of PM_2.5_, nitrogen oxides (NO–NO_2_–NOx), carbon monoxide (CO), sulfur dioxide (SO_2_), ozone (O_3_), atmospheric visibility, and solar radiation were measured, together with the meteorological parameters such as ambient air temperature (T), relative humidity (RH), and air pressure (P). The PBL heights (from 8:00 to 18:00) were measured by a micro-pulse lidar. The chemical composition of aerosol particles was measured by an Aerodyne Compact Time-of-Flight Aerosol Mass Spectrometer, which provides the mass concentrations of sulfate (SO_4_^2−^), nitrate (NO_3_^−^), ammonium (NH_4_^+^), chloride (Cl^−^), and organics. The aircraft measurement was conducted by a Yun-12 airplane, and an aerosol particle Passive Cavity Aerosol Spectrometer Probe (PCASP) instrument was used to measure the aerosol particles during the flights. The meteorological parameters such as ambient air temperature, relative humidity, and air pressure were measured during flights. Two numerical models were used in this study, including a state-of-the-art radiation transfer model (the Tropospheric Ultraviolet-Visible Model (TUV)) and an empirical model for calculating the PBL heights. More details about the measurements and model configurations are described in the Supplementary Information.

## Electronic supplementary material

Supplementary Information
