# Supplementary material for: Severe Pollution in China Amplified by Atmospheric Moisture
Source: Sci Rep. 2017 Nov 17;7:15760. doi: 10.1038/s41598-017-15909-1 (PMC5693870; doi:10.1038/s41598-017-15909-1)
Supplement: Supplementary file 1 — Supplementary Information [file 41598_2017_15909_MOESM1_ESM.pdf]

Supporting Information

Severe Pollution in China Amplified by Atmospheric Moisture

Xuexi Tie[1,2,3,4], Ru-Jin Huang[1,5,6*], Junji Cao[1*], Qiang Zhang[7], Yafang Cheng[8*], Hang Su[8], Di Chang[8], Ulrich Pöschl[8], Thorsten Hoffmann[9], Uli Dusek[10], Guohui Li[1], Douglas R. Worsnop[11] and Colin D. O'Dowd[6]

[1]Key Laboratory of Aerosol Chemistry and Physics, Institute of Earth Environment, Chinese Academy of Sciences, Xi'an 710061, China

[2]State Key Laboratory of Loess and Quaternary Geology, Institute of Earth Environment, Chinese Academy of Sciences, Xi'an 710061, China

[3]Center for Excellence in Urban Atmospheric Environment, Institute of Urban Environment, Chinese Academy of Sciences, Xiamen 361021, China

[4]National Center for Atmospheric Research, Boulder, CO, USA

[5]Laboratory of Atmospheric Chemistry, Paul Scherrer Institute (PSI), 5232 Villigen, Switzerland

[6]School of Physics and Centre for Climate and Air Pollution Studies, National University of Ireland Galway, Galway, Ireland

[7]Beijing Weather Modification Office, Beijing, China

[8]Multiphase Chemistry Department, Max Planck Institute for Chemistry, Mainz, Germany

[9]Institute of Inorganic and Analytical Chemistry, Johannes Gutenberg University of Mainz, Duesbergweg 10-14, 55128 Mainz, Germany

[10]Centre for Isotope Research (CIO), Energy and Sustainability Research Institute Groningen (ESRIG), University of Groningen, The Netherlands

[11]Aerodyne Research, Inc., Billerica, MA, USA

Correspondence and requests for materials should be addressed to R.-J.H

(rujin.huang@ieecas.cn) or J.C. (jjcao@ieecas.cn) or Y.C. (yafang.cheng@mpic.de).

Sampling information. The measurement was conducted from 19 Nov. 2012 to 15 Jan. 2013 in the urban area of Beijing (the capital city of China) at Baolian meteorological station (39°56′N, 116°17′E). The distance of the station from nearby major roads is about 400 m, and the region surrounding the measurement station is a mainly residential district without large point sources of $PM_{2.5}$. In order to clearly understand the horizontal and vertical transport, the measurements include the surface and vertical measurements. Aircraft and lidar measurements are applied for the measurements of PBL and aerosol vertical profiles. The measured variables include atmospheric visibility, the mass concentration of $PM_{2.5}$, gaseous pollutants (SO_2, NOx, CO, O_3), planetary boundary layer (PBL) height, air temperature, relative humidity (RH), pressure, wind speed, and wind direction.

The mass concentration of $PM_{2.5}$ was measured by an Element Oscillating Microbalance (TEOM; Thermo Scientific Co., USA) instrument, with the model of R&P 1400a, which is tapered with a 2.5 μm cyclone inlet and an inlet humidity control system. The instrument was housed in an air-conditioned room and was operated with a hydrophobic filter material to reduce the humidity of incoming sampled air. The sample stream was preheated before entering the mass transducer, and semi-volatiles and water were not measured. The filter loading percentage and flow rates of TEOM were checked once a week, and the filter was replaced when the filter loading percentage was greater than 30%[1].

A micro-pulse lidar (the model of MPL-4B, Sigmaspace Co., USA) was employed to study the evolution of PBL. The pulse repetition frequency of the MPL is 2500 Hz, with a wavelength of 532 nm of the laser beam. The peak value of the optical energy of laser beam is 8 μJ. The pulse duration was set to 100 ns, and the pulse interval was set to 200 ns, corresponding to a spatial resolution of 30 meters. The PBL height is detected by the sharp changes of the Lidar signals. The analysis of the PBL height by Quan et al[2]. suggests that the MPL instrument is only suitable for measuring the PBL height during the daytime. As a result, we will limit our analysis of PBL height from 8:00 to 18:00.

The gas chemical species were measured by several instruments. Nitrogen oxides (NO–NO_2–NO_x) were measured with a chemiluminescent trace level analyzer (TEI;

Model 42iTL). The analyzer has a detection limit of 0.025 ppbv. Carbon monoxide (CO) was measured by an enhanced CO analyzer, with the Model 48iTL. The instrument uses a gas filter correlation technology, with a detection limit of 0.04 ppmv. Sulfur dioxide (SO_2) was measured by a pulsed UV fluorescence analyzer (TEI; Model 43 i-TLE). The detection limit for the analyzer is 0.05 ppbv for 2-min integration with a precision of about 0.20 ppbv. Ozone (O_3) was measured with a UV photometric analyzer (TEI; Model 49iTL), with a detectable limit of 0.05 ppbv.

Atmospheric visibility was observed by a PWD20 instrument (Vaisala Co., Finland). The detecting range of visibility is from 10 to 20,000 meters. Other important meteorology variables (such as air temperature, relative humidity (RH), pressure, wind speed, and wind direction) were measured by WXT-510 (Vaisala Co., Finland).

The chemical composition of aerosol particles was measured by an Aerodyne Compact Time-of-Flight Aerosol Mass Spectrometer (C-ToF-AMS). The sampling time resolution was 2 minutes. The measured composition of particles included sulfate (SO_4^{2-}), nitrate (NO_3^-), ammonium (NH_4^+), chloride (Cl^-), and organics.

Vertical distributions of water vapor and PM. The aircraft measurement was conducted by a Yun-12 airplane, with an averaged flight speed of 200 km hr^{-1}. The vertical profiles were measured at Sha-He (SH) Airport in the north edge of the Beijing City. Over the airport, the aircraft first climbed up to about 3.6 km, and then spirally descended to the surface. The detailed vertical distributions were measured during the descend flights at different altitudes. Measurements were made with an external inlet of the Model 1200 passive Isokinetic Aerosol Sampling Inlet (BMI, Brechtel Manufacturing Inc.). The Inlet was designed to deliver 150 lpm of sample flow with 100 m s^{-1} air speeds and to transmit particles with diameters between 0.01 and 6 μm with a >95% efficiency. Aerosol particles ranging from 0.1 to 3.0 μm in diameter with 15 unequally size bins were measured by a Passive Cavity Aerosol Spectrometer Probe (PCASP) instrument, with a one-second interval of sampling resolution. The in-situ meteorological parameters such as ambient air temperature (T), relative humidity (RH), and air pressure (P) were measured during flights. The detailed information of the instruments is described by Zhang et al.[3].

Fig. S1 showed the in-situ aircraft measured vertical profiles of PBL heights, aerosol particles, RH, and water vapour during six flights in 2005 and 2006. The results showed that there was a similarity between the vertical distributions of water vapour and aerosol particles, suggesting that water vapour was also strongly constrained by PBL heights. During the shallow PBL period, water vapour depressed by PBL height, leading to high concentrations of water vapour and RH values.

Long-term statistics of AOD vs. surface $PM_{2.5}$. During the past 13 years (2001-2013), significant seasonal variation in the aerosol optical depth (AOD) can be found with the highest values in summer and spring and the lowest in winter (Fig. S2*A*, daily data source: AERONET, AErosol RObotic NETwork, http://aeronet.gsfc.nasa.gov). The seasonal patterns of surface $PM_{2.5}$ concentration are shown in Fig. S2*B* (daily data source: Ministry of Environmental Protection of the People's Republic of China, http://datacenter.mep.gov.cn/report/air_daily/air_dairy.jsp). Although the AOD is lowest during wintertime, the surface $PM_{2.5}$ is on the other hand quite high, which is opposite to the summer. Given the AOD is integrations of light extinction of particles in the total atmospheric column, the difference between the seasonal variations of AOD and $PM_{2.5}$ implies that the pollutants during wintertime have been "trapped" in a much lower altitude, compared with in summer. This leads to severe and frequent haze events in the surface layer in winter.

Fig. S3 shows the column AOD against surface $PM_{2.5}$ in summer and winter. In summer, they are almost linearly positive correlated with very few outliers, which is probably due to the well-developed boundary layer and vertical mixing. However, in winter, there are more extreme cases (marked by red outliers). The slope of AOD-versus-$PM_{2.5}$ in winter is not as steep as in summer, which means that although the column AOD in winter is not as high as in summer, the surface $PM_{2.5}$ concentration can be extremely high. Specifically, to reach the same level of surface $PM_{2.5}$, e.g., 100 $\mu g\ m^{-3}$ (marked by the vertical dashed lines in Fig. S3), the vertical AOD in winter is on average ~2.5 at 440 nm, which is much lower than in summer (~4 at 440 nm). This again demonstrates that low boundary layer, weak winds, and stagnant weather conditions during wintertime block the dispersal of pollutants, resulting in the accumulation of pollutants near the ground surface.

The radiation model (TUV). The calculation of the effect of aerosols on solar radiative forcing is conducted by using a radiative transfer model (the Tropospheric Ultraviolet-Visible Model (TUV)) developed by Madronich and Flocke[4]. TUV is a state-of-the-art radiation transfer model and is widely used by the scientific community. The code and the description of the model are available from http://www.acd.ucar.edu/TUV. The model calculates spectral irradiance, spectral actinic flux, and photo-dissociation rates (J values) for the wavelength range between 121 and 750 nm. The particle effects on solar actinic flux are mainly dependent on particle absorbing and scattering properties.

In this study, the default TUV model is modified for the calculation of particle effect on the solar radiation on the surface. The default model uses a vertical profile of aerosol optical depth (AOD), which represents a background clean air condition. In order to represent the aerosol condition in the polluted Beijing region, the AOD is calculated by the aerosol concentration measured in the P4 episode (as shown in Fig. 1*A*). The calculation is made based on the following steps and assumptions.

First, the aerosol single scattering albedo is assumed to be 0.85. The value is consistent to the composition measurements[5], in which a large amount of aerosol particles are scattering particles, such as sulfate, nitrate, ammonium, and organic carbon aerosols. Second, the AOD vertical profile is calculated based on the measurement of $PM_{2.5}$ mass concentrations. Based on long-term statistics, Wu et al.[6] indicated that the RH values of Wintertime in Beijing were on average lower than 30-40%. Accordingly, we assume that the non-linear polynomial fit in Fig. S3*B*, in general, represents the correlation between surface $PM_{2.5}$ concentration and vertical AOD under the dry conditions. The surface $PM_{2.5}$ concentrations during P4 were not exceeding 200 μg m^{-3} and still within the range that vertical AOD is nearly linear correlated with surface $PM_{2.5}$. By applying this empirical parameterization, the AOD under dry conditions can be roughly estimated from the measured surface $PM_{2.5}$ during P4. According to the aircraft measurements of this region[2], aerosol particles are generally constrained within the PBL height. As a result, the vertical profiles of aerosol can be assumed to be a constant inside PBLH, and greatly reduced outside of the PBLH. In the solar radiation calculation, we assumed that the AOD is a constant value below 1 km.

Finally, to take aerosol hygroscopic growth into account, we refer to our observation of hygroscopic growth of ambient aerosol in Beijing in the winter of 2005 (the only data of Beijing winter ambient aerosol by Meier et al.)[7]. For RH between 30%-90% (30-90 in percent), the expression is

$$D_w(\mathrm{RH}) = D_d \times (1.25 + 1.77\times10^{-5}\times\mathrm{RH} + 7.75\times10^{-5}\times\mathrm{RH}^2)$$

in which D_w(RH) is the diameter of wet particles under different RHs (in percent) and D_d is the diameter of dry particles. The conversion between the mass to number concentrations are made by the assumption that the aerosol number distribution is following the Gaussian distribution[7], with a mean radius and standard deviation 0.15 and 2.20 µm, respectively. An amplification factor is applied to the dry AOD accordingly. From the daily radio sounding data (at 8:00 and 20:00 local time), one can see that during the haze event, the vertical distribution of RH within the first 1 km (PBL or higher) was rather constant, as high as on the surface or even slightly increase in some cases (Fig. S1). In the current calculation, we thus treat the RH vertically homogeneous within the PBL (≤ 1 km).

The empirical model for the calculation of PBL height. The calculation of the effect of aerosols and water vapor on solar radiative forcing and PBL height is conducted by using an empirical model developed by Nozaki[8]. The Nozaki model is expressed by

$$H = \frac{121}{6}(6-P)(T-T_d) + \frac{0.169P(U_z+0.257)}{12f\ln(Z/z_0)}$$

where H is the PBLH, P is the Pasquill stability level. Defined by six classes from A to F in terms of increasing order from very unstable (A), moderately unstable (B), slightly unstable (C), neutral (D), slightly stable (E) to moderately stable (F)[9]. T is the surface air temperature, T_d is the surface air dew-point temperature, U_z is the mean wind speed (m s^{-1}) at height of Z (Z=10 m), f is the Coriolis parameter (s^{-1}), z_0 is the surface roughness length (0.5 m in this study), Ω is the (7.29×10^{-5} rad s^{-1}). T, T_d, and P are factors considering thermodynamic mechanism, while U_z and z_0 are taking turbulence and dynamic mechanisms into account.

According to Wallace and Hobbs[10], in the equation, T-Td can be replaced by (100-

RH)/5. As a result, the calculation of PBL height is strongly dependent upon the following parameters, such as the Pasquill stability level, relative humidity (RH), and wind speed at 10 m. In this calculation, the measured wind speeds were used in the calculation, ranging from 0.4 m s^{-1} to 0.9 m s^{-1}. In the "dry" case, the RH values were low, with a constant value of 30%. In the "wet" case, the measured RH values were used in the calculation, ranging from 39% to 83%. The Pasquill stability levels were dependent upon the effect of aerosols on solar radiation. In the "dry" case, the Pasquill stability levels ranged from 2 to 4, while in the "wet" case, the atmosphere was more stable, ranging from 2 to 5.

References

1. Zhao, X. *et al.* Seasonal and diurnal variations of ambient PM2.5 concentration in urban and rural environments in Beijing. *Atmos. Environ.* **43,** 2893–2900 (2009).
2. Quan, J. *et al.* Evolution of planetary boundary layer under different weather conditions, and its impact on aerosol concentrations. *Particuology* **11,** 34–40 (2013).
3. Zhang, Q., Ma, X., Tie, X., Huang, M. & Zhao, C. Vertical distributions of aerosols under different weather conditions: Analysis of in-situ aircraft measurements in Beijing, China. *Atmos. Environ.* **43,** 5526–5535 (2009).
4. Madronich, S. & Flocke, S. in *Environmental Photochemistry* **2 / 2L,** 1–26 (Springer Berlin Heidelberg, 1999).
5. Sun, J. *et al.* Highly time- and size-resolved characterization of submicron aerosol particles in Beijing using an Aerodyne Aerosol Mass Spectrometer. *Atmos. Environ.* **44,** 131–140 (2010).
6. Wu, Z. *et al.* Particle number size distribution in the urban atmosphere of Beijing, China. *Atmos. Environ.* **42,** 7967–7980 (2008).
7. Meier, J. *et al.* Hygroscopic growth of urban aerosol particles in Beijing (China) during wintertime: a comparison of three experimental methods. *Atmos. Chem. Phys.* **9,** 6865–6880 (2009).
8. Nozaki, K. Y. *Mixing Depth Model Using Hourly Surface Observations Report 7053, USAF Environmental Technical Applications Center. Report* (1973).
9. Pasquill, F. The Estimation of the Dispersion of Windborne Material. *Meteor. Mag.* **90,** 33–49 (1961).
10. Wallace, J. M. & Hobbs, P. V. *Atmospheric Science, Second Edition*. (Elsevier, 2005). doi:10.1016/b978-0-12-732951-2.50004-1

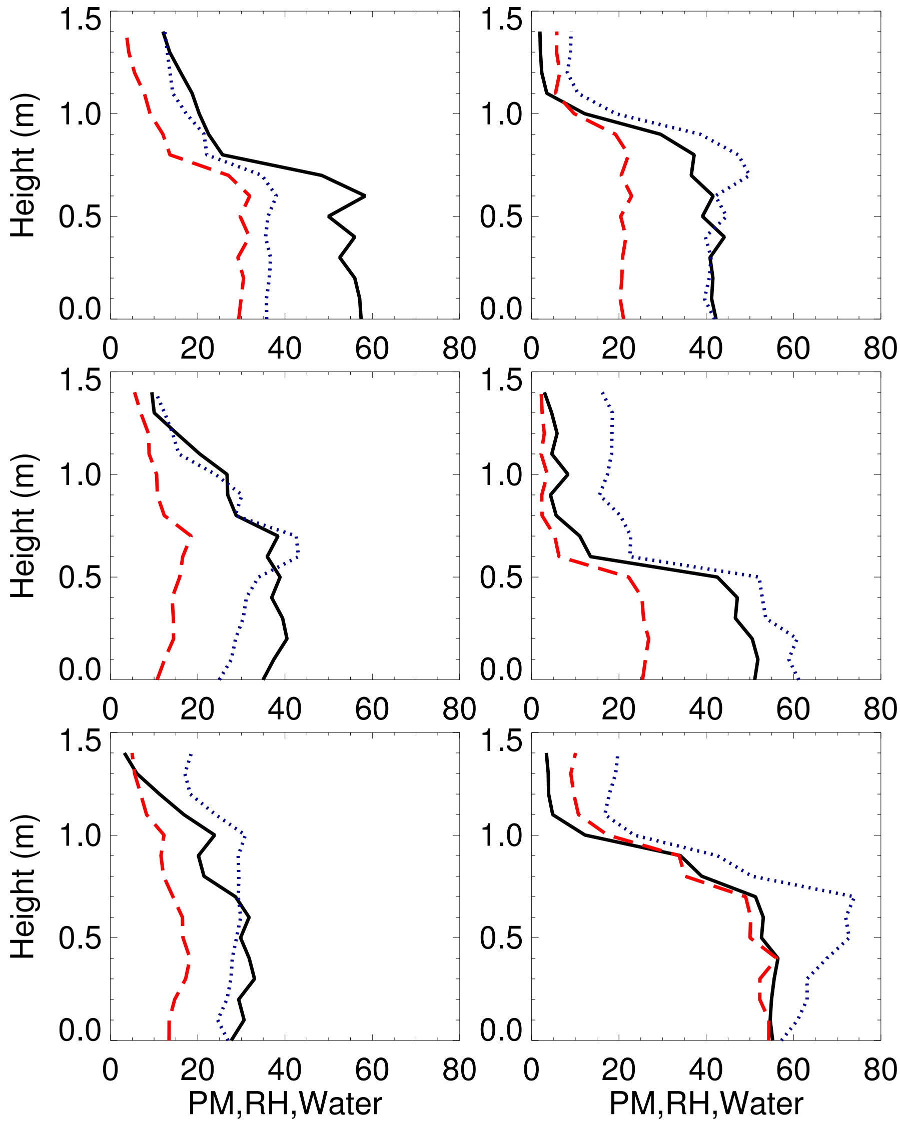

Fig. S1 Vertical distributions of aerosol particle number concentration (# cm^{-3}), water content (g m^{-3}), and RH (%) measured by aircraft during March and April in 2005-2006 in Beijing. The black-solid, blue-dot, and red-dash lines represent $PM_{2.5}$ concentration, water content, and RH, respectively. The aerosol number concentration and water content are multiplied by the factors of 5.0 $\times 10^{-3}$, and 2.0 $\times 10^{5}$, respectively. The grey lines show the locations of the top of PBL. The use of 2005-2006 vertical profiles are due to the fact that it is a lack of vertical profiles during 2013 winter, and the physics of the PBL depressing on aerosol/vapour does not change yearly.

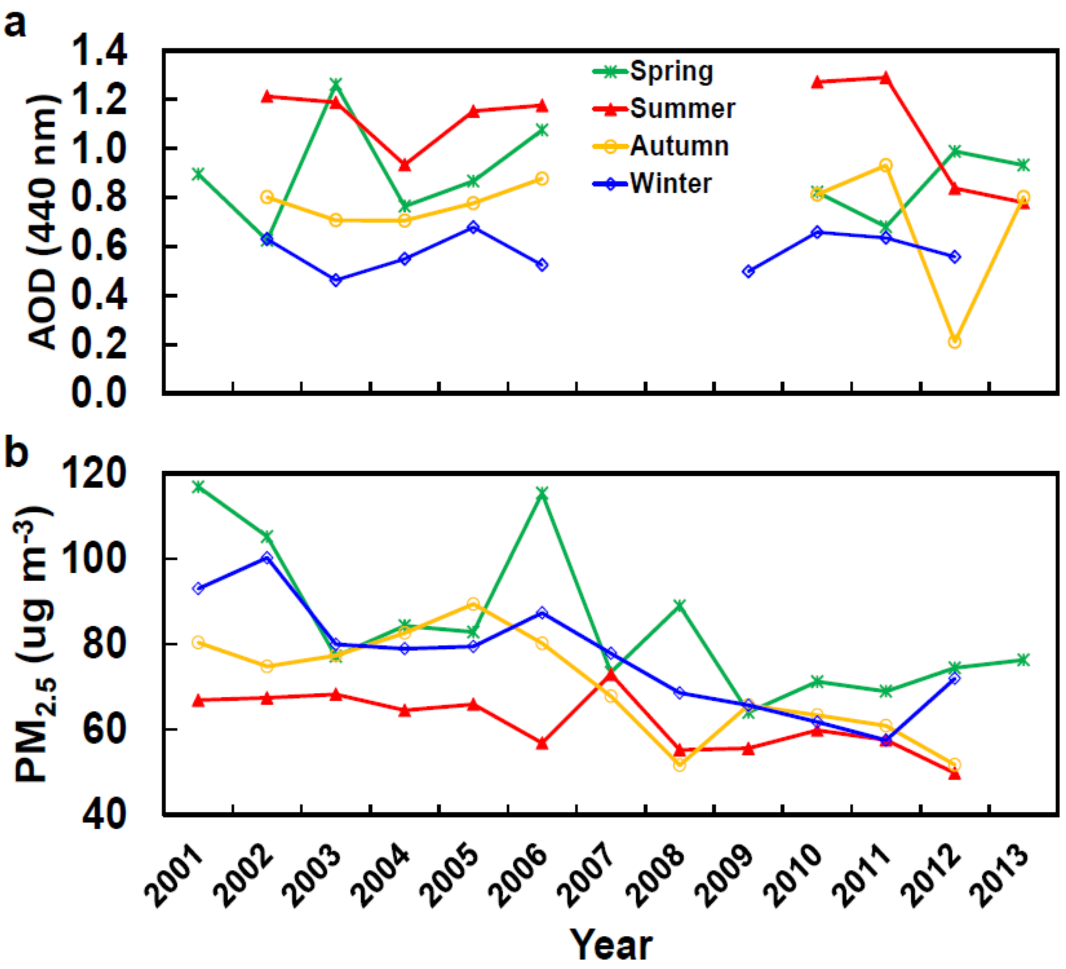

Fig.S2 Seasonal variations of aerosol optical depth (*A*) and surface $PM_{2.5}$ concentrations (*B*) in Beijing from 2001 to 2013. The aerosol optical depth (AOD) data are presented at a wavelength of 440 nm and data during Jan. 2007-Nov. 2009 are missing.

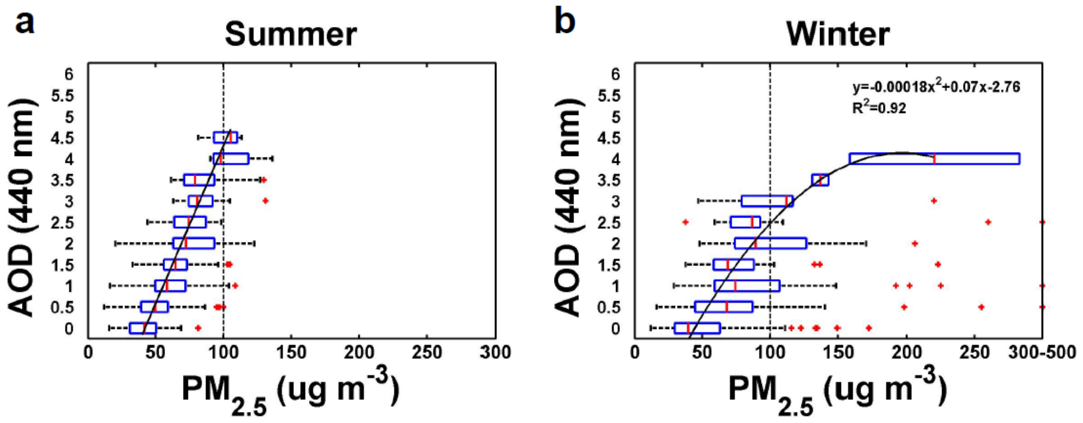

Fig. S3 Relationship between surface $PM_{2.5}$ and aerosol optical depth (AOD) for summer (*A*) and winter (*B*) in Beijing over the years of 2001-2013. The central red mark in each blue box is the median value, the edges of the blue box are the 25th and 75th percentiles, the dashed whiskers extend to cover 99.3% of the data points, and outliers are plotted as a red plus. The black line in (*A*) is a linear fit, while in (*B*) it is a polynomial fit.
